# Uncovering RNA Editing Sites in Long Non-Coding RNAs

**DOI:** 10.3389/fbioe.2014.00064

**Published:** 2014-12-05

**Authors:** Ernesto Picardi, Anna Maria D’Erchia, Angela Gallo, Antonio Montalvo, Graziano Pesole

**Affiliations:** ^1^Department of Biosciences, Biotechnologies and Biopharmaceutics, University of Bari, Bari, Italy; ^2^Institute of Biomembranes and Bioenergetics, Bari, Italy; ^3^RNA Editing Laboratory, Oncohaematology Department, IRCCS Ospedale Pediatrico Bambino Gesù, Rome, Italy; ^4^Department of Molecular Biology, Faculty of Medicine, University of Cantabria, Santander, Spain; ^5^University Hospital Marqués de Valdecilla, Santander, Spain

**Keywords:** RNA editing, RNA-Seq, ncRNA, transcriptome, A-to-I editing, long non-coding RNA, lncRNA

## Abstract

RNA editing is an important co/post-transcriptional molecular process able to modify RNAs by nucleotide insertions/deletions or substitutions. In human, the most common RNA editing event involves the deamination of adenosine (A) into inosine (I) through the adenosine deaminase acting on RNA proteins. Although A-to-I editing can occur in both coding and non-coding RNAs, recent findings, based on RNA-seq experiments, have clearly demonstrated that a large fraction of RNA editing events alter non-coding RNAs sequences including untranslated regions of mRNAs, introns, long non-coding RNAs (lncRNAs), and low molecular weight RNAs (tRNA, miRNAs, and others). An accurate detection of A-to-I events occurring in non-coding RNAs is of utmost importance to clarify yet unknown functional roles of RNA editing in the context of gene expression regulation and maintenance of cell homeostasis. In the last few years, massive transcriptome sequencing has been employed to identify putative RNA editing changes at genome scale. Despite several efforts, the computational prediction of A-to-I sites in complete eukaryotic genomes is yet a challenging task. We have recently developed a software package, called REDItools, in order to simplify the detection of RNA editing events from deep sequencing data. In the present work, we show the potential of our tools in recovering A-to-I candidates from RNA-Seq experiments as well as guidelines to improve the RNA editing detection in non-coding RNAs, with specific attention to the lncRNAs.

## Introduction

Massive transcriptome sequencing through high-throughput platforms has defiantly revealed that in mammals the vast majority of transcripts have little protein-coding potential (Djebali et al., 2012). Despite previous thoughts, large-scale projects like ENCODE have clearly demonstrated that more than 80% of mammalian genomes is transcribed and comprises numerous genes for non-coding RNAs (Consortium, 2012). These studies have shown that RNA is not only an essential intermediate in the flux of genetic information from DNA to proteins, but rather is a molecule involved in a plethora of fundamental cellular processes. Transfer RNAs (tRNAs) and ribosomal RNAs (rRNA), for instance, are essential components of translational machinery and highly abundant in all living cells. Small non-coding RNAs (sncRNAs) as small nuclear RNAs (snRNAs) or small nucleolar RNAs (snoRNAs) play relevant roles in alternative splicing and in guiding RNA chemical modifications (Jacquier, 2009). Additional sncRNAs as microRNAs (miRNAs), small interfering RNAs (siRNAs), and piwi-interacting RNAs (piRNAs) are highly conserved and associated with transcriptional and post-transcriptional gene silencing through specific base pairing with their target genes (Jacquier, 2009; Luteijn and Ketting, 2013).

Besides the different families of sncRNAs, a large proportion of the mammalian transcriptome includes RNA transcripts not coding for proteins, longer than 200 nucleotides, and defined as long non-coding RNAs (lncRNAs) (Fatica and Bozzoni, 2014). Such RNAs are poorly conserved, often polyadenylated, unstable, present in few copies and with biological roles not yet fully understood (Fatica and Bozzoni, 2014). Recent functional investigations, however, are shedding light on their functional activities and data on well-characterized lncRNAs have recently shown that such molecules have the ability to control the gene expression program at multiple levels (Wapinski and Chang, 2011). Of note, lncRNAs seem to be implicated in post-transcriptional gene regulation or in transcriptional gene silencing at epigenetic level through chromatin remodeling (Bernstein and Allis, 2005; Whitehead et al., 2009).

Virtually the entire collection of primary RNA transcripts, including the ncRNA fraction, can undergo post-transcriptional modifications as alternative splicing or RNA editing. In particular, RNA editing is widespread in the human transcriptome and involves mainly the deamination of adenosine (A) to inosine (I), recognized as guanosine (G) by all cell molecular machineries (Levanon et al., 2004). The family of adenosine deaminase acting on RNA (ADAR) proteins, characterized by the presence of double-stranded RNA binding domains (RBDs), is responsible for the deamination of specific or multiple adenosines depending on dsRNA secondary structures (Nishikura, 2010).

In human as well as in other mammals, RNA editing contributes to increase the transcriptome complexity expanding the repertoire of coding and non-coding RNAs with profound functional consequences. Indeed, RNA editing modifications may alter codons and generate or destroy splice sites so modulating alternative splicing events and influence the dynamics of constitutive splice sites (Nishikura, 2010) with a final tuning of gene expression (Nishikura, 2010; Pullirsch and Jantsch, 2010). RNA editing is indispensable to preserve cell homeostasis and its deregulation in human has been linked to a variety of neurological/neurodegenerative disorders and cancer (Gallo and Locatelli, 2011).

In recent years, massive sequencing of RNA (RNA-Seq) has enabled the study of entire transcriptomes at single nucleotide resolution offering the unique opportunity to explore and investigate at large scale post/co-transcriptional modifications due to RNA editing (Picardi et al., 2010). Genome wide screenings in human have revealed that hundred thousands editing sites exist. Indeed the current specialized RADAR database (a comprehensive A-to-I RNA editing database) annotates over 1.4 million A-to-I changes (Ramaswami et al., 2012; Ramaswami and Li, 2014). Of these, the vast majority (~96%) is located in repetitive Alu elements (Ramaswami and Li, 2014) that comprise 11% of the human genome (having a copy number exceeding 1 million copies) and are transcribed and particularly abundant within introns and untranslated regions of mRNAs (UTRs) of RNA molecules. When located in opposite orientation, two Alu elements can fold into stable secondary structures which are a suitable target for ADAR activity (Savva et al., 2012).

Also lncRNAs are potential substrates for ADARs because of their ability to fold into specific secondary structures endowed of numerous functional properties as a consequence of their interaction with proteins or other RNAs. Indeed, lncRNAs secondary structures are quite versatile even though hard to predict by conventional computational tools. Consequently, the pattern of RNA editing could be largely dynamic making difficult investigations aimed to elucidate the final functional effects of A-to-I changes on lncRNAs.

The bioinformatic prediction of RNA editing changes by RNA-Seq data is tricky with several challenges as the discrimination of true RNA editing sites from genome-encoded SNPs and technical artifacts caused by reverse-transcription, sequencing, or read-mapping errors (Ramaswami et al., 2012). Indeed, reliable RNA editing candidates require DNA-Seq support from the same sample/individual from which RNA has been sequenced and the use of several stringent filters.

Recently, we have developed and released REDItools, a specialized bioinformatics package conceived to work with NGS data (RNA-Seq for deep RNA sequencing and DNA-Seq for massive genomic DNA sequencing) and implementing a variety of filters to provide reliable sets of RNA editing sites overcoming main sequencing biases (Picardi and Pesole, 2013). REDItools run on main unix/linux operating systems and can handle pre-aligned reads from whatever sequencing platform in the standard BAM format (they do not employ information from optional SAM/BAM fields).

In the present work, we describe a computational strategy to reliably detect A-to-I alterations in human lncRNAs through deep sequencing experiments. We apply our method to high-coverage public DNA-Seq and RNA-Seq dataset from human cell line GM12878 making use of REDItools and lncRNA transcript annotations from NON-CODEv4.1, one the most updated and comprehensive databases for lncRNAs (Xie et al., 2014).

## Materials and Methods

### Data sets

Our workflow was tested on lymphoblastoid cell line GM12878 whose genome and RNA have been deeply sequenced. Pre-aligned DNA-Seq reads in BAM format were downloaded from the 1000 Genomes Project web page^1^ and re-headed using the Picard ReplaceSamHeader.jar tool.

RNA-Seq reads, instead, were downloaded as FASTQ files from UCSC genome browser.^2^ They consist of 499.4 million reads in two replicates.

### Quality check and genome mapping of RNA-Seq data

RNA-Seq quality was checked by FASTQC^3^ and trimming of low quality read ends was performed by trim_galore^4^ (phred cut-off was fixed to 20) excluding reads with a final length lower than 50 bases. A custom python script was used to remove reads containing low complexity regions or long stretches of unknown bases (Ns). STAR (Dobin et al., 2013) program with default parameters was used to identify reads mapping onto known rRNA annotations obtained from UCSC genome browser. Ribosomal reads were removed from next analysis step using an in house script (available upon request).

Cleaned RNA-Seq reads were aligned onto the human reference genome (hg19 assembly) using GSNAP program (main parameters were -s known-splicesites -E 1000 -n1 -Q -O --nofails -A sam --split-output = outputGsnap) providing a set of known splice sites from UCSC, RefSeq, Ensembl, and Gencode (Wu and Nacu, 2011). Unique and concordant paired-end alignments were converted to BAM format and used for downstream analyses. Duplicated reads were marked using the Picard MarkDuplicates.jar tool.

The REDItoolBlatCorrection.py script, included in the REDItools release, was applied to generate a list of reads mapping on multiple genome locations (default parameters were used).

### RNA editing calling

RNA editing candidates in lncRNAs were detected using the REDItoolDnaRna.py script that is part of REDItools package (Picardi and Pesole, 2013). LncRNA transcript annotations were downloaded from NON-CODEv4 database (v4.1 including 145,331 entries) (Xie et al., 2014).

## Results

RNA-Seq is the *de facto* standard approach to investigate complex eukaryotic transcriptomes as well as co/post-transcriptional modifications occurring right inside. It is particularly helpful for comprehensively identifying RNA editing sites in combination with whole genome dataset to avoid false candidates due to single nucleotide polymorphisms (SNPs). Pre-aligned reads from DNA-Seq and RNA-Seq experiments constitute the input for our REDItools that implement extensive filters to mitigate sequencing biases, thus providing reliable lists of A-to-I RNA editing candidates.

### Workflow for RNA editing detection

The main critical issue in the detection of RNA editing sites by NGS data is the mapping of RNA-Seq and DNA-Seq reads onto the reference genome that, in turn, relies on the type and quality of input data. Indeed, low quality reads lead to numerous non-canonical RNA editing sites while very short reads (<50 nucleotides) are prone to misalignments (Oshlack and Wakefield, 2009).

Before the alignment onto the reference genome, RNA-Seq reads are checked using the FASTQC program^3^ that provides basic statistics about the global quality of the experiment and allows the discovery of sequencing anomalies. For example, standard RNA-Seq libraries show altered nucleotide composition (the first 6–10 read positions) due to the use of random hexamers in the library preparation. Also, RNA-Seq reads could include overrepresented sequences due to adaptors, contaminants, or rRNAs not completely depleted. In addition, RNA-Seq experiments from degraded RNA may lead to high read duplication rates (Adiconis et al., 2013).

**Figure 1:**
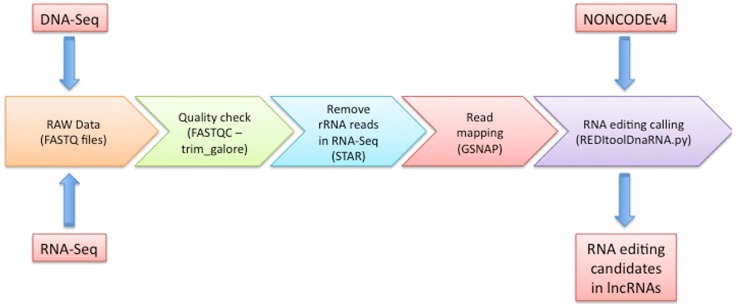
**Graphical overview of our computational methodology**. In this figure, we show all steps that should be followed to predict potential RNA editing sites in human lncRNA transcripts. Details are discussed in the main text.

As depicted in Figure 1, our workflow starts with a FASTQC run to carefully check the quality of input experiments and design the next trimming step through the *trim_galore* utility^4^. Independently of FASTQC results, we removed low quality regions at 3′ ends of reads using a phred cut-off value of at least 20 and we excluded reads containing low complexity regions or long stretches of unknown nucleotides (Ns). Optionally, we add a quick step to eliminate reads showing high similarity to rRNAs by means of STAR program (Dobin et al., 2013) and custom scripts (available upon request).

After the quality assessment and an accurate data preprocessing, RNA-Seq reads are aligned onto the reference genome using GSNAP (Wu and Nacu, 2011), providing a non-redundant collection of known splice sites extracted from well-established databases as UCSC, RefSeq, Ensembl, and Gencode (Harrow et al., 2012). Although a plethora of mapping tools have been released, we preferred to use GSNAP since resulted one of the best performing aligners in a recent systematic evaluation of spliced alignment programs for RNA-Seq data (Engstrom et al., 2013). In addition, we demonstrated that realignment of RNA-Seq reads by GSNAP increased the detectability of RNA editing sites (Picardi and Pesole, 2013).

Following the mapping, GSNAP generates nine separate output files in the standard SAM format, one for each alignment type (concordant, halfmapping, paired and unpaired, etc.). Only unique and concordant alignments (in case of paired-end reads) are retained and used for downstream RNA editing calling.

An accurate detection of A-to-I editing events relies also on the type of input RNA-Seq reads. Optimal results are expected from experiments generating ultra-deep paired and stranded reads of at least 75 nucleotides. The type of RNA-Seq reads is particularly important for lncRNAs since many of them are natural antisense transcripts or produced from intronic regions of protein coding genes either in the sense or antisense direction. In addition, RNA-Seq libraries should be sequenced at high coverage since lncRNAs are generally expressed at low levels.

Although GSNAP works accurately, misalignment errors may occur. The mismapping effect can be mitigated realigning reads carrying mismatches by the classical Blat algorithm through an *ad hoc* script included in REDItools (REDItoolBlatCorrection.py). Such script identifies reads prone to mismapping and collects them in specific lists, ready to be inspected by main REDItools programs.

### RNA editing calling by REDItools

Uncovering RNA editing in lncRNAs is based on the REDItoolDnaRNA.py script in which single RNA editing modifications are identified by comparing pre-aligned RNA-Seq and DNA-Seq reads from the same sample/individual. Briefly, the script explores genomic positions site by site and applies several filters taking into account the coverage depth, the base quality score, the mapping quality, the bases supporting the variation, the type of substitution and its frequency, and changes in homopolymeric regions (≥5 bases) or in intronic sequences surrounding known splice sites. If stranded RNA-Seq data are provided, the script can infer the strand for each position mitigating biases due to antisense transcription or mapping errors and can facilitate the A-to-I detection in lncRNAs. In the meantime, REDItoolDnaRNA.py interrogates also DNA-Seq alignments to exclude potential genomic SNPs. In addition, the script can work on specific genomic regions providing a valid set of coordinates in the GTF format.

### RNA editing in human lncRNAs

The above-described workflow has been applied to publicly available DNA-Seq and RNA-Seq data from human lymphoblastoid cell line GM12878. RNA-Seq data (polyA+) were obtained from the ENCODE project. Libraries were strand-specific and deeply sequenced with Illumina HiSeq2000 in two biological replicates, resulting in 235.8 and 263.7 million paired-end 76-base sequencing reads, respectively^2^. DNA-Seq data, instead, were provided as pre-aligned reads by the 1000 Genomes Project in BAM format^1^. The genomic DNA of GM12878 was sequenced at 44× coverage, allowing accurate genotype calls.

All transcriptomic reads were mapped onto the complete human genome using GSNAP in combination with a large repertoire of known splice sites. Resulting unique and concordant paired-end alignments were submitted to REDItoolDnaRNA.py as well as lncRNA transcript annotations from NON-CODE (v4.1, 145,331 entries), an integrated knowledge database dedicated to non-coding RNAs (excluding tRNAs and rRNAs) (Xie et al., 2014).

On the whole, we identified 11,726 potential RNA editing events supported by at least 10 DNA-Seq reads in the NON-CODE lncRNA transcript collection. Of these, we discarded only 227 positions annotated as genomic SNPs in dbSNP (release 138). The remaining 11,499 sites were annotated using the RepeatMask table from UCSC and NON-CODE transcripts (the complete list is available as Supplementary Material).

**Figure 2:**
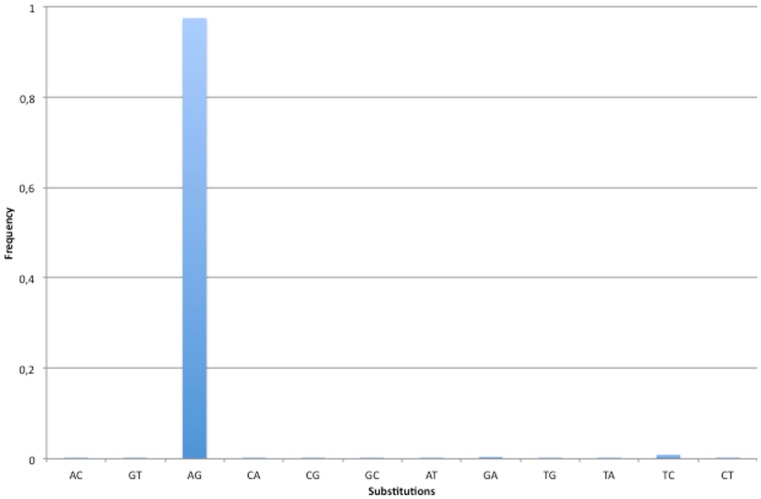
**Base substitutions observed in human lncRNAs**. The specificity of our methodology has been valuated looking at base substitutions in the set of predicted RNA editing events. Since A-to-I is the most frequent RNA editing event in human and I is commonly interpreted as G by cellular molecular machineries, the A-to-G change is expected to be the prominent substitution. As shown in figure, 97% of base changes in the predicted set of RNA editing events are A-to-G substitutions. All other changes have substitution frequencies lower than 1%.

Our screen for RNA editing in lncRNAs achieved high specificity (Figure 2). Indeed, 97.45% of all detected changes were A-to-G mismatches while the second most frequent nucleotide substitution was T-to-C, with only 0.92% of the total number of editing sites (106/11,499). However, in 91 out of 106 T-to-C modifications the REDItoolDnaRNA.py script was not able to correctly infer the strand, most likely due to sequencing errors or concomitant expression of both strands at comparable levels. We think that several of these T-to-C changes may be genuine RNA editing events.

The majority of A-to-I modifications (86% – 9,682/11,206 unique A-to-G changes) were identified in Alu repeat regions while 1,140 resided in repetitive non-Alu regions (mostly long and short interspersed elements and long terminal repeats) and only 384 in non-repetitive regions. These findings are in accordance with other genome-wide computational screens in which a large fraction of RNA editing sites (> 90%) is located in Alu repetitive elements (Ramaswami et al., 2012; Bazak et al., 2014). The observed RNA editing pattern suggests that also in lncRNAs, Alu base pairing is predominant even though its functional role is yet elusive.

**Figure 3:**
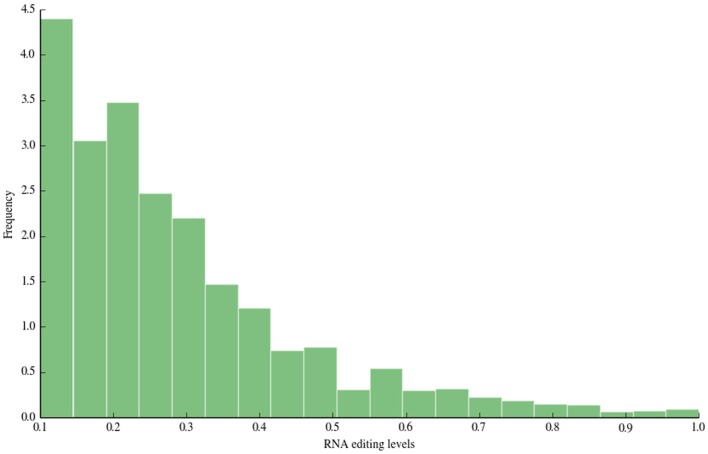
**RNA editing levels**. In this figure, we depict the distribution of RNA editing levels. The vast majority of detected sites show low editing levels (<0.5), in accordance with previous large-scale studies.

The distribution of RNA editing levels is shown in Figure 3. Like other previous studies, the vast majority of detected A-to-I changes showed low RNA editing levels (<0.5).

Almost all edited Alus were in intronic regions of lncRNAs while only 1913 A-to-I changes were located in exons. Excluding Alu elements, very few positions (104 sites) were found in non-repetitive regions of lncRNAs. In this reduced pool of sites, we observed several RNA editing clusters that may indicate the presence of secondary RNA structures. Such RNA editing sites may have important functional roles altering the secondary structure of lncRNAs preventing or promoting interactions with proteins or other RNAs.

The 11,206 unique A-to-G changes fell in 1649 lncRNA gene loci (3374 lncRNA transcripts) and, of these, a substantial number occurred in intervening sequences. According to the NON-CODE database in which lncRNA genes are classified into four categories depending on their genomic location in relation to protein-coding genes (antisense, intergenic, sense exonic, and sense non-exonic), we valuated the distribution of edited lncRNA genes among these four categories. A consistent amount (62%) of lncRNA genes was predominantly in the sense exonic category and only 51 (3%) belonged to sense non-exonic grouping. The number of lncRNA genes cataloged as antisense and intergenic was roughly equivalent, being 267 and 289, respectively.

We finally compared our list of RNA editing changes with that identified in a previous study based on the same NGS dataset by using a slighting different methodology (Ramaswami et al., 2012). We found overlap for 10,898 sites (97%) indicating high specificity of our computational strategy and improved sensitivity over past bioinformatic methods.

## Discussion

Large-scale projects, such as the ENCODE (Encyclopedia of DNA Elements), have markedly revealed the pervasiveness of genome transcription (Consortium, 2012). Nearly 60% of human genome encodes transcripts that lack protein-coding capacity but with a potential role in multiple biological processes (Djebali et al., 2012). Among them, a particular attention has focused on a class of transcripts indicated as lncRNAs, generally defined as RNAs longer than 200 nucleotides (Fatica and Bozzoni, 2014). Although lncRNAs are poorly conserved, unstable, and present in few copies, they have been implicated in transcriptional regulation of protein-coding gene (Fatica and Bozzoni, 2014).

In addition to transcriptional complexity of eukaryotic genomes, the transcriptome landscape is further complicated by co/post-transcriptional mechanisms as alternative splicing and RNA editing (Djebali et al., 2012; Bazak et al., 2014). In particular, RNA editing may play relevant biological roles also at level of lncRNAs (Mallela and Nishikura, 2012). In human, the majority of RNA editing modifications is constituted by A-to-I conversions carried out by the ADAR enzymes (Ramaswami and Li, 2014). These proteins have the ability to target secondary RNA structures and deaminate specific adenosines located inside (Nishikura, 2010). Due to their secondary structures, lncRNAs are expected to be potential targets of ADARs with specific functional effects such as preventing or promoting interactions with proteins or other RNAs. The importance of studying RNA editing modifications in lncRNAs is mainly justified in pathological conditions in which editing events may be connected with alteration of lncRNA expression/function.

Nowadays lncRNAs and RNA editing can be profiled at single nucleotide resolution through NGS technologies (Picardi et al., 2010; Ramaswami et al., 2012; Ding et al., 2014). The massive transcriptome sequencing, indeed, facilitates the identification of lncRNAs as well as the detection of putative RNA editing events (Picardi et al., 2010). However, the computational prediction of RNA editing changes by RNA-Seq is not trivial due to technical artifacts (sequencing or read-mapping errors) and genomic information from same samples/individuals is required to discriminate true RNA editing sites from SNPs (Ramaswami et al., 2012).

To uncover the RNA editing landscape using NGS data, we have recently developed the package REDItools that includes specific scripts to investigate RNA editing starting from matched RNA-Seq and DNA-Seq data or RNA-Seq data alone (Picardi and Pesole, 2013). In the present work, we introduce a computational methodology devoted to the detection of RNA editing events in human lncRNAs, demonstrating in the meantime the suitability of our REDItools as a versatile package for screening RNA editing candidates in NGS data.

We tested our pipeline on DNA-Seq and RNA-Seq data from human lymphoblastoid cell line GM12878 using 145,331 lncRNA transcripts from NON-CODE database (Xie et al., 2014). Compared with previous computational pipelines (Ramaswami et al., 2012), our methodology achieved high specificity and improved sensitivity, as already shown in Picardi and Pesole (2013). Indeed, more than 97% of detected RNA editing changes were A-to-G mismatches mainly distributed in Alu repeated regions.

The majority of edited lncRNA genes were in the sense exonic category meaning that RNA editing target lncRNA genes were in overlap with known protein coding genes and in the same orientation. In such cases, since lncRNAs and overlapping protein coding transcripts share the same strand, the assessment of RNA editing membership, lncRNA or coding transcript, is very hard. Further checks taking into account the expression levels of involved genes and transcripts are extremely needed before claiming novel discoveries.

Although the computational detection of RNA editing events in NGS data is not yet completely optimized, our REDItools are the only available software to explore the RNA editing landscape in complete transcriptomes. Given the explosion of NGS technologies in genomic research, REDItools and derived methodologies, as the one described in this work, will be indispensable to characterize RNA editing in novel experimental conditions as well as in human disorders.

## Conflict of Interest Statement

The authors declare that the research was conducted in the absence of any commercial or financial relationships that could be construed as a potential conflict of interest.

## Supplementary Material

The Supplementary Material for this article can be found online at http://www.frontiersin.org/Journal/10.3389/fbioe.2014.00064/abstract

## Footnotes

^1^http://www.1000genomes.org

^2^http://hgdownload.cse.ucsc.edu/goldenpath/hg19/encodeDCC/wgEncodeCshlLongRnaSeq/

^3^http://www.bioinformatics.babraham.ac.uk/projects/fastqc/

^4^http://www.bioinformatics.babraham.ac.uk/projects/trim_galore/
